# Significant promotion of porous architecture and magnetic Fe_3_O_4_ NPs inside honeycomb-like carbonaceous composites for enhanced microwave absorption†

**DOI:** 10.1039/c8ra00913a

**Published:** 2018-05-23

**Authors:** Shengshuai Gao, Qingda An, Zuoyi Xiao, Shangru Zhai, Zhan Shi

**Affiliations:** Faculty of Light Industry and Chemical Engineering, Dalian Polytechnic University Dalian 116034 China zhaisrchem@163.com anqingdachem@163.com; State Key Laboratory of Inorganic Synthesis and Preparative Chemistry, College of Chemistry, Jilin University Changchun 130012 China

## Abstract

Carbonaceous composites with tailored porous architectures and magnetic Fe_3_O_4_ components derived from walnut shells were fabricated by a solvothermal method and used as effective microwave absorbers. The porous composites were obtained by two carbonization processes at different temperatures and an etching process using potassium hydroxide. The introduction of a developed porous architecture inside the resulting materials distinctly improved the microwave absorption performance. Moreover, investigations revealed that the Fe_3_O_4_ nanoparticles were chemically bonded and uniformly decorated on the porous framework without aggregation. Owing to the combined advantages of the lightweight conductive biochar-like porous framework with a favorable dielectric loss and Fe_3_O_4_ nanoparticles with magnetic loss features, these newly fabricated porous carbonaceous composites exhibited excellent microwave absorption performance. A reflection loss (RL) of −51.6 dB was achieved at a frequency of 13.6 GHz. Besides, the effective absorption (below −10 dB) bandwidth reached 5.8 GHz (from 11.9 to 17.7 GHz) at an absorber thickness of only 2 mm. These results indicated that this type of porous Fe_3_O_4_–biochar composite derived from biomass substances and prepared *via* an easy-to-handle process can be considered as attractive candidates for the design and manufacture of high-efficiency microwave-absorbing materials.

## Abstract 2

A novel type of Fe_3_O_4_/WPC-600 with a tailored porous architecture and magnetic components has been facilely fabricated for significantly enhanced microwave absorption performance.

## Graphical abstract

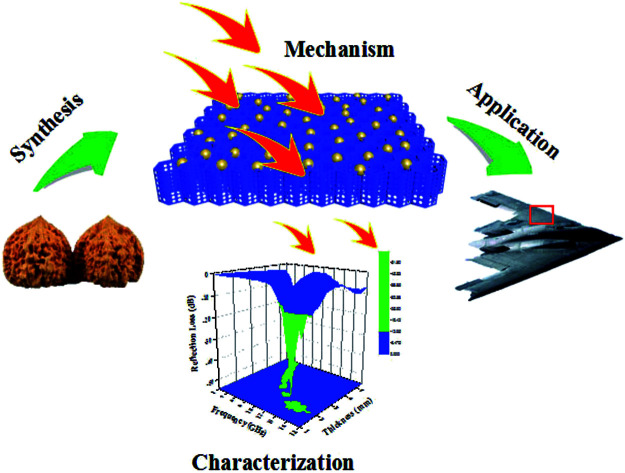

## Introduction

With the rapid advances in information technology, electronic devices are becoming smarter and smaller and growing in number every day. People are enjoying the benefits of advanced technologies but at the same time are disturbed by them. Electromagnetic interference (EMI) resulting from the expansion in the use of electronic devices will have a detrimental influence on military applications and civilian fields. More importantly, the increase in electromagnetic pollution has a harmful impact on human health. Therefore, the requirement for high-performance electromagnetic-wave-absorbing materials to solve EMI problems has been given a high value during recent years.^1–8^

Theoretically, the performance of absorbing materials is determined by their complex permeability (*μ*_r_ = *μ*′ − j*μ*′′) and permittivity (*ε*_r_ = *ε*′ − j*ε*′′). Hence, traditional efforts normally classified microwave-absorbing materials into two categories, namely, magnetic loss materials such as Fe, Co, Ni and alloys^9–12^ and dielectric loss materials such as CuS, SiC, MnO_2_ and conducting polymers.^13–16^ Unfortunately, their high density greatly limits their potential applications despite their strong absorbing properties. Conveniently, to address this issue many carbonaceous materials such as carbon nanotubes (CNTs), carbon filaments, carbon fibers and chemically derived graphene have been used as composite materials for electromagnetic-wave absorbers owing to their light weight, flexibility, high thermal conductivity, excellent mechanical properties and high electrical conductivity.^17–20^ However, most of these composites still have many disadvantages; for example, one-dimensional CNTs and two-dimensional graphene have a tendency to undergo restacking and aggregation during the preparation process, which possibly leads to a distinct reduction in available properties. Moreover, the processing methods of these carbon-based microwave-absorbing materials are relatively complicated and incur high costs. In this regard, the investigation of a low-cost controllable preparation method and the development of a new type of related absorber with greatly enhanced electromagnetic-wave absorption ability are urgently in demand.

More recently, biochars, which are derived from biological organisms, have been extensively researched and widely applied in the capture of carbon dioxide, battery anodes, adsorption of organic pollutants and supercapacitor electrode materials owing to their various inherent characteristics, for example: (1) biochar materials are abundant, do not harm the environment and are easy to obtain; (2) biochar materials possess intrinsic large pores and natural large specific surface areas; and (3) biochar materials have numbers of oxygen-containing functional groups, which can possibly facilitate the formation of chemical bonds with functional groups of modified materials.^21–24^ Most importantly, the characteristics of biochar materials would undergo some remarkable improvements upon activation with KOH, because considerable numbers of mesopores would be formed inside the activated materials. At this point, if biochar materials were used as microwave absorbers, microwave energy would be effectively attenuated by interfacial polarization relaxation loss owing to the strengthened interfacial polarization induced by solid–void interfaces. Therefore, biochar-derived materials have been regarded as promising candidates for high-performance microwave absorbers. For instance, Qiu *et al.*^25^ reported that porous biomass carbon was a lightweight and effective microwave absorber, which was mainly due to its dielectric loss; however, the contribution of the magnetic loss mechanism that can be provided by magnetic components was not employed. Actually, many kinds of oxygen-containing functional groups on the surface of biomass-derived carbons are suitable for modifying with a magnetic material to improve impedance matching, which could distinctly improve the microwave absorption performance. Amongst various magnetic species, Fe_3_O_4_ nanoparticles have received extensive attention owing to their unique properties, such as high chemical stability, high thermal stability and a moderate saturation magnetization value (*M*_S_).^26,27^ As a consequence, what would happen upon the combination of biochar materials with Fe_3_O_4_ nanoparticles to form microwave-absorbing materials? Thus far, the investigation of controllable preparation processes for this type of hybrid materials with enhanced microwave absorption performance has rarely been reported.

Here, in continuation of our research interest in the design of high-performance adsorbents/absorbers for emerging pollutants,^28–30^ a new type of honeycomb-like carbonaceous composite with tailored porous architectures and magnetic Fe_3_O_4_ nanoparticles was prepared from walnut shells *via* a controllable *in situ* solvothermal method. The walnut shells were treated by two carbonization processes, of which the first carbonization was achieved at 400 °C and the second process was finished at 600 °C, during which etching with KOH was simultaneously conducted. Then, porous carbonaceous composites decorated with Fe_3_O_4_ nanoparticles for significantly enhancing the microwave absorption performance were facilely prepared. Testing of the microwave absorption performance was carried out in detail on the resulting samples, and the relationship between the preparation conditions and absorption performance was established according to various characterization results. Finally, a synergistic effect between the porous architectures and magnetic species inside the as-fabricated carbonaceous composites was demonstrated.

## Experimental

### Materials

Walnut shells were purchased from Xiyu Meinong Co. Ltd. Potassium hydroxide (KOH) and hydrochloric acid (HCl) were purchased from Kermel. Iron chloride hexahydrate (FeCl_3_·6H_2_O) and ethylene glycol (CH_2_OH)_2_ were purchased from Sinopharm Chemical Reagent Co. Ltd. All the chemical reagents used in this work were of analytical grade and were used without further purification.

### Synthesis of porous carbon architecture derived from walnut shells

Porous carbon was synthesized as described in a previous work.^25^ In detail, walnut shells were crushed into particles (about 10 mm) and then washed three times with deionized water (20 °C) and dried at 80 °C. The dried walnut shells (10 g) were calcined in a tube furnace at 400 °C for 2 h under a nitrogen (N_2_) atmosphere at a heating rate of 10 °C min^−1^, and then the as-prepared samples were cooled in the furnace. The as-prepared substance was then etched in 30 mL of an aqueous solution of KOH (10 mol L^−1^) and stirred for 10 h at room temperature. After being dried at 80 °C, the material was calcined at 600 °C for 2 h under an N_2_ atmosphere at a heating rate of 10 °C min^−1^, and then the as-prepared materials were cooled in the furnace. Finally, the carbon material was thoroughly washed with 1 mol L^−1^ HCl solution and deionized water until neutral and then dried at 80 °C. The resulting porous carbon derived from walnut shells was named as WPC-600. For comparison, walnut shells without the etching process were denoted as WC-600.

### Synthesis of Fe_3_O_4_/WPC-600 composites

**Scheme 1:**
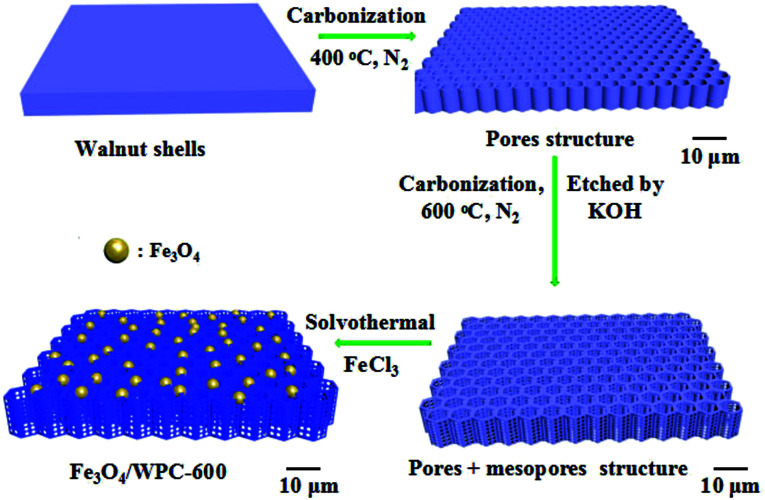
Demonstration of fabrication process used for Fe_3_O_4_/WPC-600 composite materials.

WPC-600 (100 mg) and FeCl_3_·6H_2_O (100 mg) were dispersed into ethylene glycol (50 mL) by powerful ultrasonic treatment for about 20 minutes until a homogeneous yellow suspension was obtained by this process. Then, NaOH (1 g) and N_2_H_4_·H_2_O (5 mL) were added to the above suspension, which was stirred for a further 20 min. Finally, the green suspension was transferred into a Teflon-lined stainless-steel autoclave in which the temperature was maintained at 200 °C for 12 h. The black product that was obtained after washing with deionized water and ethanol several times was referred to as Fe_3_O_4_/WPC-600. The products were named as 0.5-Fe_3_O_4_/WPC-600 and 2-Fe_3_O_4_/WPC-600 when the mass of FeCl_3_·6H_2_O was 50 mg and 200 mg, respectively. The manufacturing process of Fe_3_O_4_/WPC-600 is illustrated in Scheme 1.

### Structural characterization

The structure and morphology of the products were characterized *via* scanning electron microscopy (SEM, Nova NanoSEM, FEI), and detailed microstructural information was revealed by high-resolution transmission electron microscopy (HRTEM, Hitachi H9000NAR), Raman microprobe spectroscopy (Thermo Fisher Scientific) and X-ray diffraction (XRD). The surface properties were studied using X-ray photoelectron spectroscopy (XPS, ESCALAB 210) and Brunauer–Emmett–Teller (BET) methods *via* nitrogen adsorption and desorption measurements. The magnetic properties of the composites were studied using a vibrating sample magnetometer (VSM, Lake Shore 7304) at room temperature.

## Results and discussion

### Characterization of as-made samples

**Fig. 1:**
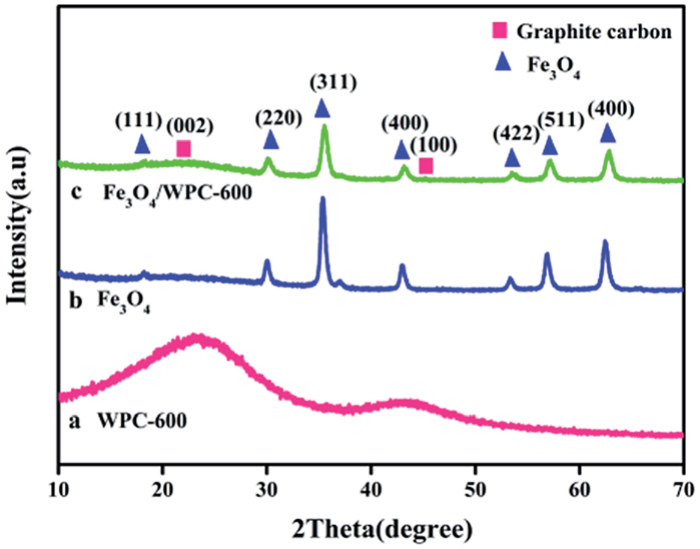
XRD patterns of (a) WPC-600, (b) pure Fe_3_O_4_ and (c) Fe_3_O_4_/WPC-600 composite.

During the solvothermal process, a quantity of Fe^3+^ was reduced to Fe^2+^ by N_2_H_4_·H_2_O. Under solvothermal treatment, Fe_3_O_4_ nanocrystals were nucleated *in situ* and grown on the surface of WPC-600, which led to the formation of Fe_3_O_4_/WPC-600. The chemical reactions can be expressed as: 4Fe(OH)_3_ + N_2_H_4_·H_2_O → 4Fe(OH)_2_ + 5H_2_O + N_2_ and 2Fe(OH)_3_ + Fe(OH)_2_ → Fe_3_O_4_ + 4H_2_O. The crystal structures of WPC-600, pure Fe_3_O_4_ and Fe_3_O_4_/WPC-600 nanocrystals were determined by XRD and are shown in Fig. 1. WPC-600 (Fig. 1a) displayed two broad diffraction peaks located at 26.1° and 43.1°, which correspond to the (002) and (100) crystal planes of graphitic carbon, respectively.^31^ For the pristine Fe_3_O_4_ nanoparticles (Fig. 1b), all the diffraction peaks can be well indexed to the face-centered cubic (fcc) spinel form of Fe_3_O_4_ (JCPDS card no. 74-0748). For Fe_3_O_4_/WPC-600 (Fig. 1c), the 2*θ* values of all the diffraction peaks can be well matched to the peak positions of the two components (pure Fe_3_O_4_ and WPC-600). No characteristic peaks due to impurities were detected, which suggested the phase purity of all the samples, and the sharpness of the peaks shows that the sample of Fe_3_O_4_/WPC-600 was highly crystalline. Nevertheless, all the corresponding areas of the peaks are less than those for the two components (pure Fe_3_O_4_ and WPC-600). This might be attributed to the formation of oxygen-containing functional groups such as carbonyl, hydroxyl and epoxy groups between the two components during the combination process, which implies that Fe_3_O_4_ might undergo chemical bonding with WPC-600. In particular, an unclear characteristic broad peak located at about 26.1° and an unobserved broad peak located at about 43.1° suggest that decoration with Fe_3_O_4_ nanoparticles could reduce the graphitization degree and make the porous carbon more disordered in the Fe_3_O_4_/WPC-600 composite.

**Fig. 2:**
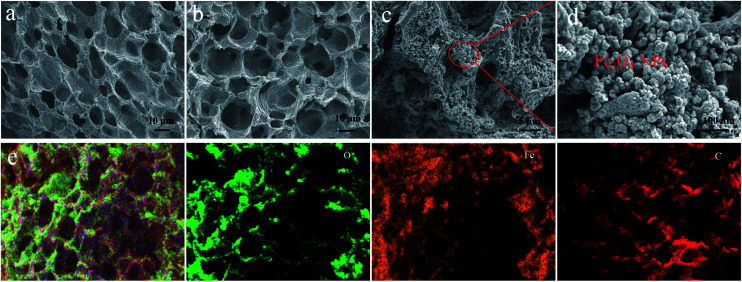
SEM images of WC-600 (a), WPC-600 (b) and Fe_3_O_4_/WPC-600 at different magnifications (c and d); EDS elemental mapping images of Fe_3_O_4_/WPC-600 (e).

The morphology and structures of the as-prepared materials were characterized by SEM and are shown in Fig. 2. It is clearly seen that all the samples (Fig. 2a–c) derived from walnut shells possess natural pore structures with pore sizes of about 10 μm regardless of whether or not the sample has been etched. This porous structure allowed KOH to flow into the interior of the samples easily, which enabled an adequate etching process. After the etching process, the surface exhibited a distinct increase in BET surface area (*S*_BET_) (Table 1), which was due to a large number of mesopores (Fig. S1†). However, the *S*_BET_ value of Fe_3_O_4_/WPC-600 decreased after the loading of Fe_3_O_4_ nanoparticles (Table 1). The main reason for the great decrease in BET surface area was that it was calculated in units of m^2^ g^−1^. In Fig. 2d and e, it is clearly shown that Fe_3_O_4_ nanoparticles were evenly distributed on the surface and natural pores of WPC-600. This composite might be beneficial for microwave absorption owing to the interfacial polarization and structural defects, whereby defect sites possibly generate an additional energy state as a result of the uneven and stepped energy levels near the Fermi level.^24^ The elemental mapping of Fe_3_O_4_/WPC-600 is shown in Fig. 2e. Obviously, the heterostructure character is confirmed by the distribution of elements, namely, O element and Fe element were homogeneously scattered in the region of C element.

**Table 1:** Specific surface area (*S*_BET_), total pore volume, and most probable pore size (*W*_peak_) of samples

Sample	*S*_BET_ (m^2^ g^−1^)	Total pore volume (cm^3^ g^−1^)	*W*_peak_ (nm)
WC-600	188.4	0.16	8.4
WPC-600	509.3	0.20	5.6
Fe_3_O_4_/WPC-600	130.4	0.10	8.9

**Fig. 3:**
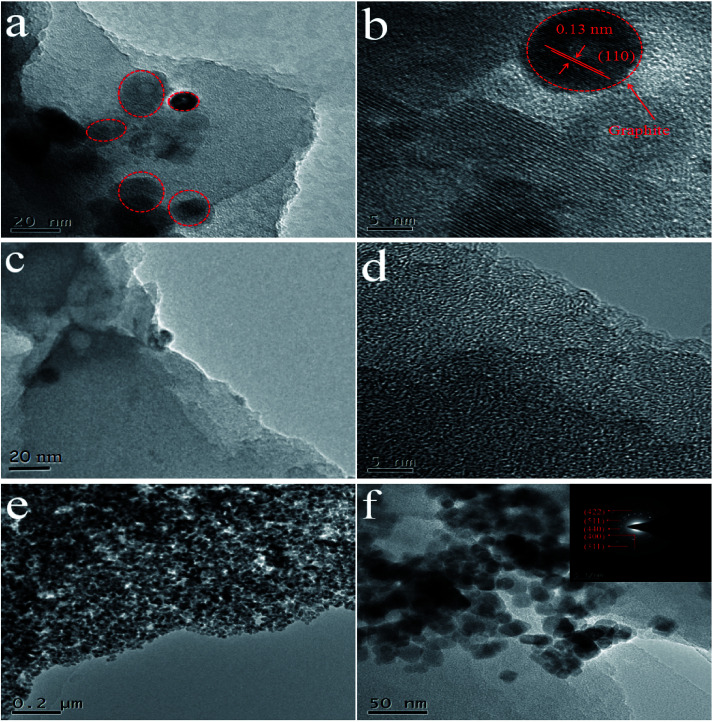
TEM images of (a and b) WC-600, (c and d) WPC-600 and (e and f) Fe_3_O_4_/WPC-600 composite at different magnifications. The inset (f) shows the SAED pattern of Fe_3_O_4_ nanocrystals in the Fe_3_O_4_/WPC-600 composite.

**Fig. 4:**
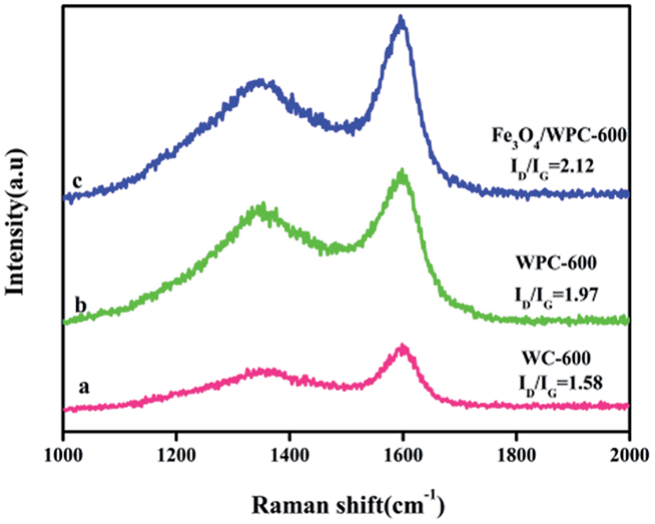
Raman spectra of WC-600 (a), WPC-600 (b) and Fe_3_O_4_/WPC-600 (c).

The morphology and microstructures of as-synthesized samples were further observed *via* TEM images. For WC-600 (Fig. 3a), it is shown that there are several relatively intact lattice fringe areas. The lattice spacing is seen more clearly in an HRTEM image (Fig. 3b), and the interplanar spacing is 0.13 nm, which corresponds to the (110) plane of hexagonal graphite.^32^ For comparison, graphite lattice fringes did not appear in a large area of WPC-600 (Fig. 3c and d), which implied that the etching process destroyed the graphite structure and made it more disorderly. TEM images at different magnifications (Fig. 3e and f) show that Fe_3_O_4_ nanoparticles were uniformly decorated on WPC-600 without aggregation and with no large vacancies. A histogram of the size distribution of Fe_3_O_4_ nanoparticles (Fig. S2†) shows that the Fe_3_O_4_ nanoparticles possessed a uniform diameter of approximately 6 nm. Moreover, the results in the histogram are also in accordance with a Gaussian distribution (standard deviation of 7%), which indicates that the Fe_3_O_4_ nanoparticles on WPC-600 were monodisperse with a narrow size distribution. The fact that no Fe_3_O_4_ nanoparticles were found outside the carbon film implies a possible chemical bonding attraction between the carbonized materials and Fe_3_O_4_ nanoparticles, which corresponds to the XRD results. The SAED pattern of an Fe_3_O_4_/WPC-600 composite is shown in the inset of Fig. 4f. The sample possessed high crystallinity, which was confirmed by bright and distinguishable diffraction rings. The diffraction rings can be indexed to the (440), (511), (422), (400) and (311) lattice planes, which illustrates the formation of the fcc structure of Fe_3_O_4_, which is in accordance with the XRD results.

In order to obtain more information on the graphitization degree of the three composites, Raman spectra were recorded. For all the composites, two characteristic bands (D-band at 1340 cm^−1^ and G-band at 1590 cm^−1^) can be clearly seen in Fig. 4. The former corresponds to the out-of-plane vibrations of sp^3^ carbon atoms in defect-rich or disordered carbon, whereas the latter represents a characteristic in-plane vibration of sp^2^-hybridized carbon atoms.^33^ The intensity ratio of the D- and G-bands (*I*_D_/*I*_G_) is related to the graphitization degree of carbon. For WC-600, WPC-600 and Fe_3_O_4_/WPC-600, the calculated *I*_D_/*I*_G_ values are 1.58, 1.97 and 2.12, respectively. Obviously, WPC-600 has a higher value of *I*_D_/*I*_G_ than WC-600, which indicates that the structure became more disorderly in WPC-600 after etching with KOH. Moreover, the value of *I*_D_/*I*_G_ increased from 1.97 to 2.13 after WPC-600 was modified with Fe_3_O_4_ nanoparticles, which implies that more defects were formed in the Fe_3_O_4_/WPC-600 samples.

**Fig. 5:**
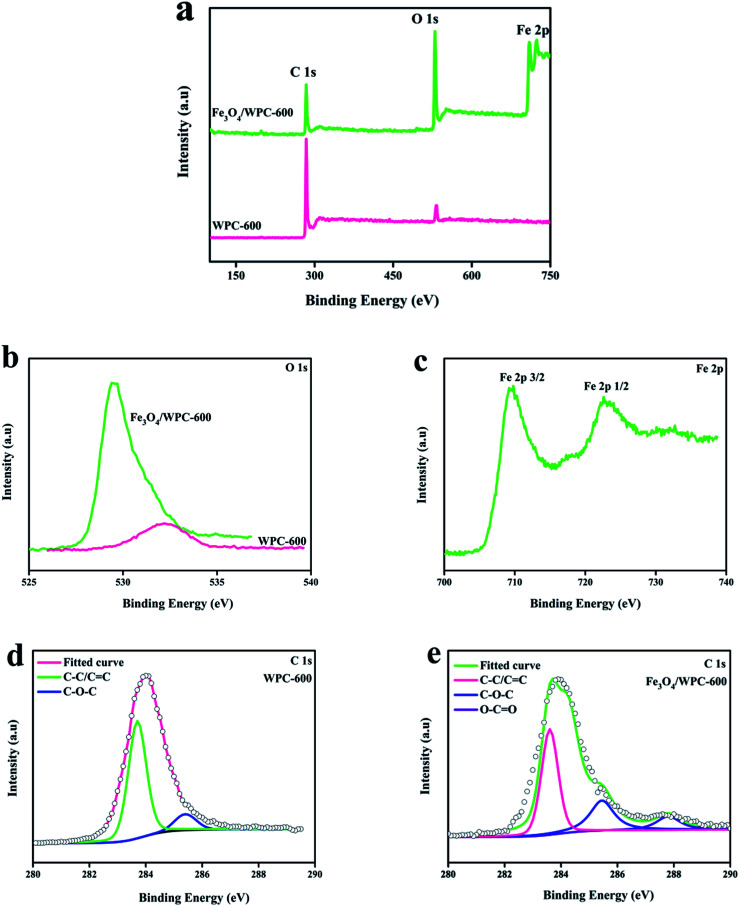
XPS spectra: (a) wide-scan spectra of WPC-600 and Fe_3_O_4_/WPC-600 composite; (b) O 1s spectra of WPC-600 and Fe_3_O_4_/WPC-600 composite; (c) Fe 2p spectrum of Fe_3_O_4_/WPC-600 composite; C 1s spectra of (d) WPC-600 and (e) Fe_3_O_4_/WPC-600 composite.

The XPS survey spectra of WPC-600 and Fe_3_O_4_/WPC-600 composites were also analyzed, in which the chemical compositions and elemental states are indicated in Fig. 5. Fig. 5a shows the wide-scan XPS spectra of WPC-600 and an Fe_3_O_4_/WPC-600 composite. The three sharp peaks at binding energies of 285.2, 530.5 and 711.6 eV correspond to C 1s, O 1s and Fe 2p, respectively. This indicates the presence of C and O elements in WPC-600 and C, O and Fe elements in the Fe_3_O_4_/WPC-600 composite. It is noted that some oxygen element is naturally present in WPC-600. To further characterize the elemental states, high-resolution XPS spectra were studied. Fig. 5b shows the high-resolution O 1s spectra, and the binding energy for the O 1s peak shifted from 532.4 eV for WPC-600 to 529.2 eV for Fe_3_O_4_/WPC-600, which was due to the presence of oxygen in Fe_3_O_4_. The high-resolution Fe 2p spectrum (Fig. 5c) of Fe_3_O_4_/WC-600 displays two characteristic peaks at 710.9 and 725.1 eV, which are associated with Fe 2p_3/2_ and Fe 2p_1/2_, respectively. It is worth noting that no satellite peak appeared at about 719.2 eV, which can exclude the presence of γ-Fe_2_O_3_ in the composite. Therefore, it can be concluded that the iron oxide in the composite was present as Fe_3_O_4_. Fig. 5d and e show the C 1s spectra of WC-600 and an Fe_3_O_4_/WPC-600 composite, respectively. In the high-resolution C 1s spectrum of WPC-600 (Fig. 5d), two deconvolved peaks at binding energies of 283.7 and 285.4 eV can be attributed to C–C/C=C and C–O–C groups, respectively. After Fe_3_O_4_ was used to modify WPC-600 (Fig. 5e), there was an additional deconvolved peak at 287.8 eV, which corresponded to O–C=O groups. The fact that the carbonized materials and Fe_3_O_4_ nanoparticles were chemically bonded was confirmed by the results of XPS.

**Fig. 6:**
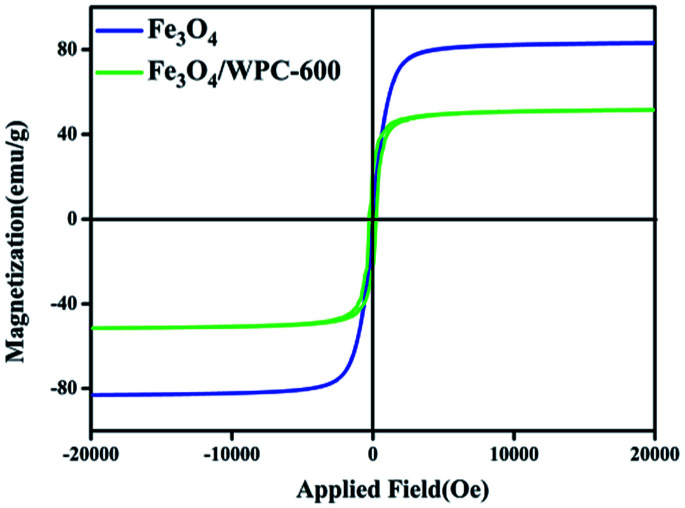
Magnetic hysteresis loops of bare Fe_3_O_4_ and Fe_3_O_4_/WPC-600 recorded at room temperature.

The magnetic properties of bare Fe_3_O_4_ and Fe_3_O_4_/WPC-600 were measured by a vibrating sample magnetometer (VSM) at room temperature. Three samples exhibited S-shaped curves with low coercivities (*H*_C_) and negligible remanence (Fig. 6), which demonstrated superparamagnetic characteristics. The values of the saturation magnetization (*M*_S_), coercivity (*H*_C_), and remanent magnetization (*M*_r_) are shown in Table 2. Obviously, the *M*_S_ value of the Fe_3_O_4_/WPC-600 composite was lower than that of bare Fe_3_O_4_, which was due to the presence of diamagnetic carbon, which reduced the corresponding parameters. Nevertheless, the *H*_C_ value of Fe_3_O_4_/WPC-600 was higher than that of bare Fe_3_O_4_. It is thus proposed that the resulting Fe_3_O_4_/WPC-600 composites may have enhanced microwave absorption properties because of a higher magneto-crystalline anisotropy energy induced by the higher coercivity.^34–36^ Moreover, the values of the conductivity *σ* of all the samples are shown in Table S1.† The results indicate that Fe_3_O_4_ can greatly improve the value of *σ*, which may be of great significance for microwave absorption.

**Table 2:** Saturation magnetization (*M*_S_), coercivity (*H*_C_) and remanent magnetization (*M*_r_) of Fe_3_O_4_ and Fe_3_O_4_/WPC-600

Sample	Saturation magnetization (*M*_S_) (emu g^−1^)	Coercivity (*H*_C_) (Oe)	Remanent magnetization (*M*_r_) (emu g^−1^)
Fe_3_O_4_	82.98	42.33	1.46
Fe_3_O_4_/WPC-600	51.47	248.64	21.82

### Electromagnetic-wave absorption properties

**Fig. 7:**
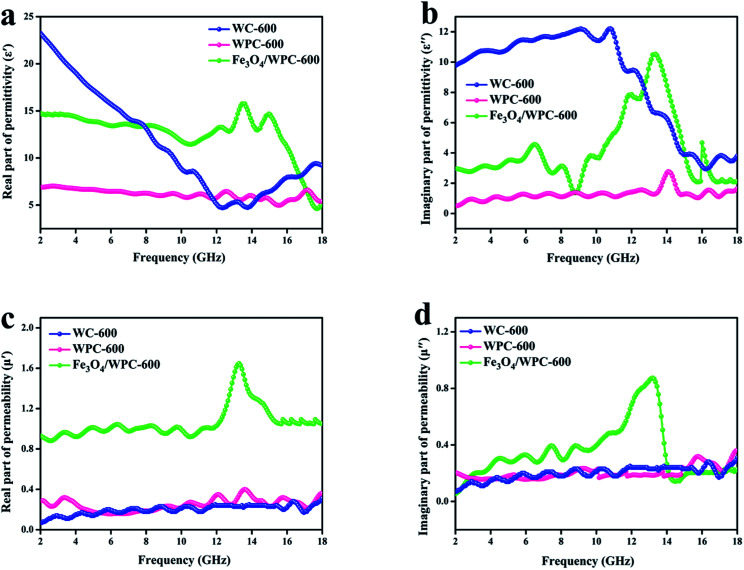
Frequency dependence of (a) real and (b) imaginary parts of complex permittivity and (c) real and (d) imaginary parts of complex permeability of the samples.

According to the theory of electromagnetic energy conversion, microwave absorption performance is determined by the relative complex permittivity (*ε*_r_ = *ε*′ − j*ε*′′) and permeability (*μ*_r_ = *μ*′ − j*μ*′′). The real parts of the complex permittivity (*ε*′) and permeability (*μ*′) represent the ability to store electrical and magnetic energy, respectively. The imaginary parts (*ε*′′ and *μ*′′) are related to the ability to dissipate electrical and magnetic energy, respectively.^7,8,12^ Hence, these electromagnetic parameters (complex permittivity and permeability) of the samples were measured and are shown in Fig. 7. As seen in Fig. 7a, the *ε*′ values of WC-600 displayed a distinct decreasing tendency from 2 to 12 GHz and ranged from 23.6 to 4.7. It can also be observed that the variation tendency in *ε*′′ (Fig. 7b) was nearly the same. Theoretically, a higher permittivity means a higher ability to store and dissipate electrical energy, and hence the sample displays better microwave absorption performance.^37^ Nevertheless, if the permittivity is much higher than the permeability (shown in Fig. 7c and d), this will result in poor impedance matching. Impedance matching requires that the values of permittivity and permeability be close to make electromagnetic waves enter the interior of materials as much as possible.^38^ Therefore, even though WC-600 has high permittivity, it might exhibit poor microwave absorption performance owing to poor impedance matching. In comparison, the *ε*′ values of WPC-600 were relatively stable with an increase in frequency from 2 to 18 GHz, with values of between 7.2 and 8.6. Obviously, the *ε*′ values of the sample decreased after etching with KOH. This phenomenon can be explained by effective medium theory (EMT).^25^ An incident wave is not sensitive to structures that are smaller than a sensing wavelength. The effective mean value (*ε*_eff_) of *ε* is determined by this property of radiation and is calculated by the Maxwell–Garnett equation, which can be expressed as follows:^39^

1 *ε*^MG^_eff_ = *ε*_1_[(*ε*_2_ + 2*ε*_1_) + 2*f*_r_(*ε*_2_ − *ε*_1_)]/[(*ε*_2_ + 2*ε*_1_) − *f*_r_(*ε*_2_ − *ε*_1_)]

where *f*_r_ is the volume fraction of voids in the effective medium and *ε*_1_ and *ε*_2_ represent the dielectric permittivity of the solid and the voids, respectively.^40^ The *f*_r_ value increased because many mesopores were created in the sample after etching with KOH. Therefore, the effective permittivity decreased according to the Maxwell–Garnett theory. After decoration with Fe_3_O_4_ nanoparticles, we can see that the *ε*′ values of Fe_3_O_4_/WPC-600 increased and ranged from 13.7 to 16.2. Fe_3_O_4_/WPC-600 had higher *ε*′ values, which might have been due to space charge polarization because of the heterogeneity of the composite material.^41^ The presence of Fe_3_O_4_ nanoparticles decorated on the surface of porous carbon leads to the generation of more space charge and its accumulation at the interface, which contributes to higher microwave absorption in the composite.^3,41^

As depicted in Fig. 7b, the *ε*′′ values of WC-600 displayed a decreasing trend from 12 GHz to 18 GHz, which implies that it might exhibit poor microwave absorption in the Ku band (12–18 GHz).^42^ For WPC-600 and Fe_3_O_4_/WPC-600, the values of *ε*′′ were similar to the *ε*′ values, which implies that the dissipation ability of these samples was coordinated with their ability to store electrical energy, which is beneficial for absorption performance. As seen in Fig. 7c and d, the *μ*′ and *μ*′′ values of the Fe_3_O_4_/WPC-600 sample exhibited broad resonance peaks over the whole frequency range (2.0–18.0 GHz), which are attributed to the natural resonance of Fe_3_O_4_.^43^ In addition, it is noticeable that the *μ*′ value of the Fe_3_O_4_/WPC-600 composite was the highest among all the samples, which indicates the highest ability to store magnetic energy. On the basis of the above discussions, the conclusion can be reached that Fe_3_O_4_/WPC-600 may possess improved microwave absorption properties owing to a high dielectric loss and magnetic loss, as well as good impedance matching.

**Fig. 8:**
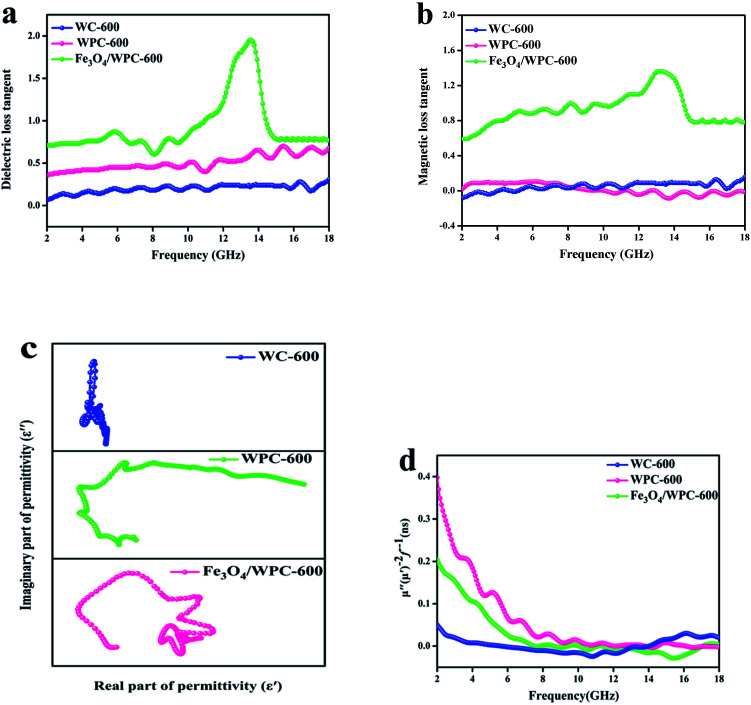
Dielectric loss tangent (a) and magnetic loss tangent (b) of as-prepared samples. Cole–Cole plots (c) and values of *μ*′′(*μ*′)^−2^*f*^−1^ (d) of as-synthesized composites.

The loss parameters can be expressed in terms of the dielectric loss tangent (tan *δ*_*ε*_ = *ε*′′/*ε*′) and magnetic loss tangent (tan *δ*_*μ*_ = *μ*′′/*μ*′). As shown in Fig. 8a, WPC-600 exhibited a higher dielectric loss tangent than WC-600 over the whole frequency range. This result indicates that the KOH etching process can greatly improve the microwave absorption properties owing to the great increase in the dielectric loss of the microwave absorber. The dielectric loss can be increased by numerous defects and mesopores in the surface of WPC-600.^44^ In addition, the dielectric loss tangent became much higher after WPC-600 was uniformly decorated with Fe_3_O_4_ nanoparticles, which was because the Fe_3_O_4_/WPC-600 composite structure introduced more nanoparticles and formed more heterogeneous interfaces, which led to interfacial polarization, dipole polarization and stronger contact relaxation at the interfaces.^45,46^ In heterogeneous dielectrics, virtual charges at the interface between two media would accumulate if the media possessed different dielectric constants and conductivities, which would result in the form of polarization known as Maxwell–Wagner polarization.^47^ The relaxation process is used to better understand the mechanism of dielectric loss of an electromagnetic absorber *via* the following equation:^48^

2 *ε*_r_ = *ε*′ + i*ε*′′ = *ε*_∞_ + (*ε*_s_ − *ε*_o_)/(1 + i*ωτ*_o_)

where *τ* is the relaxation time, *ω* is the frequency, and *ε*_s_ and *ε*_∞_ denote the static permittivity and relative dielectric permittivity at the high-frequency limit, respectively. According to the above eqn (2), *ε*′ and *ε*′′ could be calculated as indicated below:

3 *ε*′ = *ε*_∞_ + (*ε*_s_ − *ε*_o_)/[1 + (*ωτ*_o_)^2^]

4 *ε*′′ = *ωτ*_o_(*ε*_s_ − *ε*_o_)/[1 + (*ωτ*_o_)^2^]

According to eqn (3) and (4), the relationship between *ε*′ and *ε*′′ could be expressed as:

5 [*ε*′ − (*ε*_s_ + *ε*_∞_)/2]^2^ + (*ε*′′)^2^ = [(*ε*_s_ − *ε*_∞_)/2]^2^

6 *ε*′ = *ε*′′/(2π*fτ*) + *ε*_∞_

From eqn (5), we can see that a plot of *ε*′ against *ε*′′ should be a single semicircle (Fig. 8c), which indicates a Debye relaxation process and is termed a Cole–Cole plot.^49^ For WPC-600 four middle-sized semicircles can be seen, followed by a straight line, which indicates that interfacial polarization occurred in defects in carbon. An obviously different plot can be observed for Fe_3_O_4_/WPC-600 (Fig. 8c), in which many large semicircles and no straight line can be found. This should be related to the heterostructure, which might shorten the relaxation time for electron oscillations.^50^ For WC-600, the lack of an obvious semicircle means that no relaxation process occurred, which corresponded to a low dielectric loss tangent.

The magnetic loss tangents of all the samples are shown in Fig. 8b. For WC-600 and WPC-600, the plots show that the magnetic loss tangent was almost zero over the whole frequency range, which suggests that the bare carbon derived from walnuts had a negligible magnetic loss. For Fe_3_O_4_/WPC-600, the values of the magnetic loss tangent were similar to those of the dielectric loss tangent, especially in the higher-frequency range, which indicates that enhanced absorption is due to the synergy of dielectric loss and magnetic loss in composites. In addition, the broad resonance band observed for Fe_3_O_4_/WPC-600 is beneficial for the enhancement of electromagnetic-wave absorption because it could induce a strong magnetic loss. The magnetic loss mainly results from domain wall displacement, magnetic hysteresis, exchange resonance, natural resonance, and the eddy current effects.^51^ As shown in Fig. 8d, natural resonance and the eddy current effect were the main reasons for magnetic loss in the magnetic loss process. The eddy current effect can be calculated as follows:^52^

7 *C*_o_ = *μ*′′(*μ*′)^−2^*f*^−1^

If the eddy current effect dominates the magnetic loss mechanism, the value of *C*_o_ should be constant when the frequency changes. However, the *C*_o_ values of all the samples decreased with an increase in frequency (2–8 GHz), which implies that natural resonance should be emphasized. From 8 to 18 GHz, the values of *C*_o_ were nearly constant, which was termed the skin effect criterion^53^ and showed that the eddy current loss mainly affects the dissipation of microwave energy in the frequency range of 8–18 GHz.

**Fig. 9:**
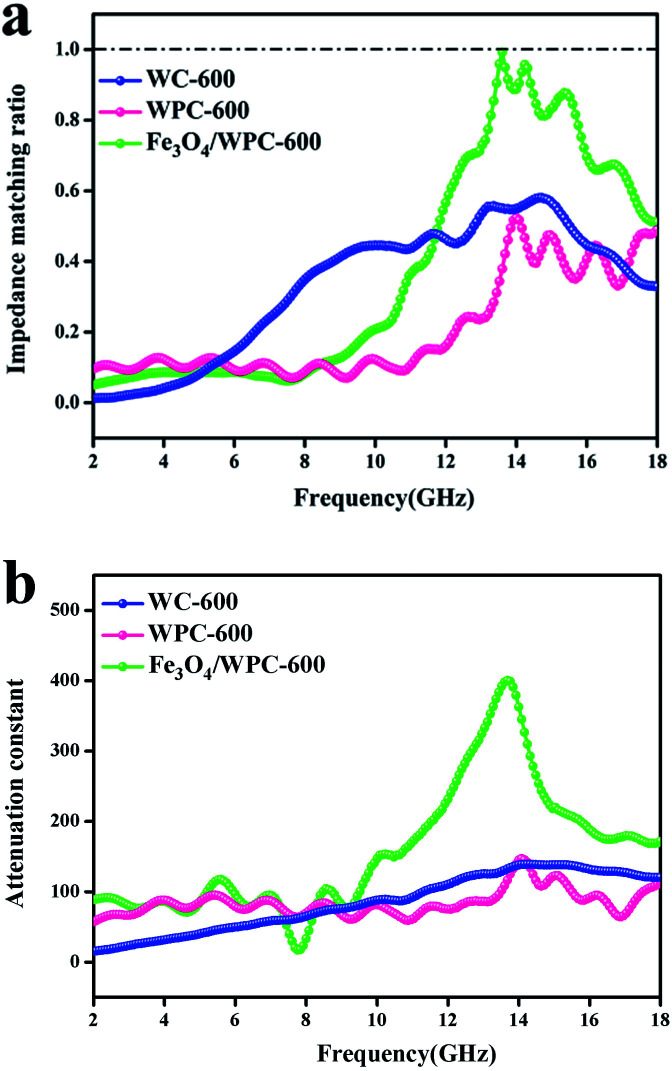
Modulus of normalized input impedance of the products at a thickness of 2.0 mm (a); attenuation constant of as-made composites (b).

According to transmission line theory, impedance matching and the attenuation constant (*α*) are quantitatively associated with the EM absorption ability. Fig. 9a shows the values of the normalized characteristic impedance (*Z* = |*Z*_in_/*Z*_o_|), which were calculated according to eqn (8) and (9):

8 *Z*_in_ = *Z*_o_(*μ*_r_/*ε*_r_)^1/2^ tan *h*[j(2π*fd*)/*c*(*μ*_r_*ε*_r_)^1/2^]

9 RL = 20 log |(*Z*_in_ − *Z*_o_)/(*Z*_in_ + *Z*_o_)|

where *f* is the frequency, *c* is the velocity of light, *d* is the thickness of the composite, and *Z*_o_ and *Z*_in_ represent the impedance of free space and input characteristic impedance, respectively. Impedance matching represents the ability of microwaves to efficiently enter the microwave absorber and be converted into thermal energy. A microwave-absorbing material would display the best performance if the value of *Z* were equal to 1. As shown in Fig. 9a, the *Z* values of Fe_3_O_4_/WPC-600 were close to 1 at 13.6 GHz, which implies that the microwave-absorbing ability of Fe_3_O_4_/WPC-600 at 13.6 GHz is excellent. In comparison, the values of *Z* for the other samples were far from 1 over the whole frequency range, which indicates poor impedance matching performance and implies that microwaves could hardly enter the absorber. The attenuation constant (*α*) is another factor that is quantitatively associated with the EM absorption ability and can be calculated using the corresponding permeability and permittivity parameters by the following equation:

10 *α* = (√2π*f*/*c*) × √[(*μ*′′*ε*′′ − *μ*′*ε*′) + √[(*μ*′′*ε*′′ − *μ*′*ε*′)^2^ + (*μ*′′*ε*′′ + *μ*′*ε*′)^2^]]

It is shown that Fe_3_O_4_/WPC-600 displayed a higher *α* value than the other samples over the whole frequency range (Fig. 9b). Therefore, we may draw the conclusion that the synergetic effect of both impedance matching and the attenuation constant of the Fe_3_O_4_/WPC-600 composite played a key role in improving the very high EM absorption properties. By analyzing the relationship between impedance matching and microwave attenuation ability, the interaction mechanism in the sample between incident waves and the absorber could be concluded to be as follows: firstly, good impedance matching conditions ensured that microwaves could enter the absorber as much as possible, and then the attenuation ability enabled the efficient absorption of incident microwaves *via* intrinsic dielectric loss and magnetic loss.

**Fig. 10:**
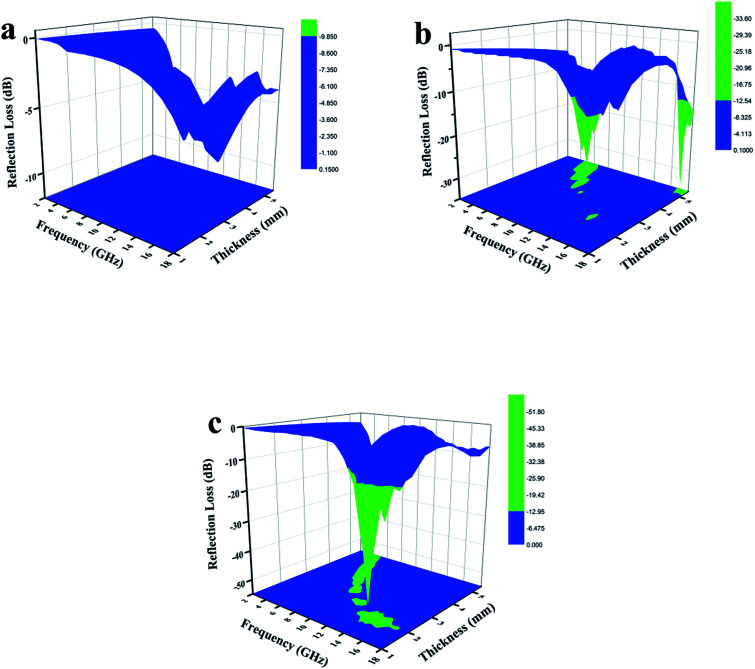
Three-dimensional images of calculated RL values of (a) WC-600, (b) WPC-600 and (c) Fe_3_O_4_/WPC-600 composites.

To further investigate the electromagnetic-wave absorption performance of the three samples, the reflection loss (RL) values of samples with different thicknesses were calculated according to eqn (8) and (9) and are shown in Fig. 10. The electromagnetic-wave absorption properties of all the composites were studied using a vector network analyzer (Agilent N5222A) in the range of 2–18 GHz. Coaxial specimens for measuring electromagnetic parameters were fabricated by mixing with 50 wt% paraffin and pressing into a cylindrical shape (*φ*_out_ of 7.0 mm, *φ*_in_ of 3.04 mm). In general, an RL value of below −10 dB means that in excess of 90% of microwaves are absorbed by the absorbing material, which is regarded as the criterion for estimating whether microwave absorbers are suitable for practical applications. WC-600 had poor microwave absorption properties because the RL values for different thicknesses were not below −10 dB (Fig. 10a). As discussed above, the reasons for the poor properties of WC-600 are its poor impedance matching and poor attenuation capacity. After the KOH etching process, which formed a more mesoporous structure, the microwave absorption performance was remarkably improved: the maximum reflection loss of WPC-600 was −33.6 dB at 6.2 GHz, and the reflection loss was below −10 dB in the range from 3.5 to 7.4 GHz, which is similar to previous reports.^25^ Interestingly, when Fe_3_O_4_ nanoparticles were decorated on porous carbon to form Fe_3_O_4_/WPC-600 composites, the microwave absorption ability was tremendously improved. Fe_3_O_4_/WPC-600 exhibited the best microwave absorption performance (Fig. 10c), and its maximum RL value reached −51.6 dB at a frequency of 13.6 GHz. Besides, the effective absorption (below −10 dB) bandwidth reached 5.8 GHz (from 11.9 to 17.7 GHz) at an absorber thickness of 2 mm and could be tuned between 8.0 and 17.7 GHz by tuning the thickness between 1.0 and 2.5 mm. All the results suggest that the Fe_3_O_4_/WPC-600 composites exhibited improved microwave absorption performance over a wide frequency range, which completely covered the X band (8–12 GHz) and the whole Ku band (12–18 GHz), which implies that they may have useful applications in military radar systems, satellite communications and weather radar owing to their precision object identification and high-resolution imaging abilities. Moreover, the same method was also carried out for two other compositions, namely, those denoted as 0.5-Fe_3_O_4_/WPC-600 and 2-Fe_3_O_4_/WPC-600, respectively. As shown in Fig. S3a_1_,† Fe_3_O_4_ nanoparticles were sparsely distributed on the surface of 0.5-Fe_3_O_4_/WPC-600. For 2-Fe_3_O_4_/WPC-600 (Fig. S3a_1_†), smaller Fe_3_O_4_ nanoparticles intensively covered the surface. The elemental mapping of 0.5-Fe_3_O_4_/WPC-600 (Fig. S3a_2_ and a_3_†) and 2-Fe_3_O_4_/WPC-600 (Fig. S3b_2_ and b_3_†) clearly shows the distribution of Fe element. The maximum reflection loss of 0.5-Fe_3_O_4_/WPC-600 (Fig. S4a†) was −35.8 dB at 5.5 GHz at an absorber thickness of 3.5 mm, and the maximum reflection loss of 2-Fe_3_O_4_/WPC-600 (Fig. S4b†) was −38.2 dB at 4.9 GHz at an absorber thickness of 4 mm. The maximum reflection losses of 0.5-Fe_3_O_4_/WPC-600 and 2-Fe_3_O_4_/WPC-600 were similar to that of WPC-600, which implies that the quantity of Fe_3_O_4_ nanoparticles had little effect in improving the microwave absorption performance. This was mainly because, regardless of the quantity of Fe_3_O_4_ nanoparticles, the synergistic effect was poor between magnetic Fe_3_O_4_ nanocrystals with magnetic loss and lightweight conductive WPC-600 with dielectric loss. The results are clearly shown in Fig. S5,† and the impedance matching ratios of 0.5-Fe_3_O_4_/WPC-600 and 2-Fe_3_O_4_/WPC-600 were far from 1. As shown in our previous discussion, poor impedance matching is not beneficial for microwave absorption performance. Therefore, it was proved that Fe_3_O_4_/WPC-600 exhibited the best microwave absorption performance owing to the synergistic effect between lightweight conductive porous carbon with dielectric loss and Fe_3_O_4_ nanoparticles with magnetic loss. Table 3 shows the microwave absorption properties of other Fe_3_O_4_–carbon-based composites.^5,13,54–56^ It can be seen that this kind of newly fabricated Fe_3_O_4_/WPC-600 composites can be regarded as promising candidates for high-performance microwave-absorbing materials.

**Table 3:** Microwave absorption properties of Fe_3_O_4_–carbon-based composites in the frequency range of 2–18 GHz, particularly within the past 5 years

Absorber	Content (wt%)	RL (min) (dB)	Thickness (mm)	Effective bandwidth (GHz) (RL < −10 dB)	Frequency range (GHz)	Ref
Fe_3_O_4_/GCs	30	−32.0	3.5	4	8–12	5
Fe_3_O_4_–RGO	50	−44.6	3.9	4.3	12.2–16.5	13
Fe_3_O_4_/SWCNHs	50	−38.8	5.8	2.2	10–12.2	54
Fe_3_O_4_–RGO	50	−44.6	3.9	4.3	12.2–16.5	55
Fe_3_O_4_/GO/CNT	30	−37.3	5.0	2.2	9.8–12	56
Fe_3_O_4_/WPC-600	50	−51.3	2.0	5.8	11.9–17.7	This work

**Fig. 11:**
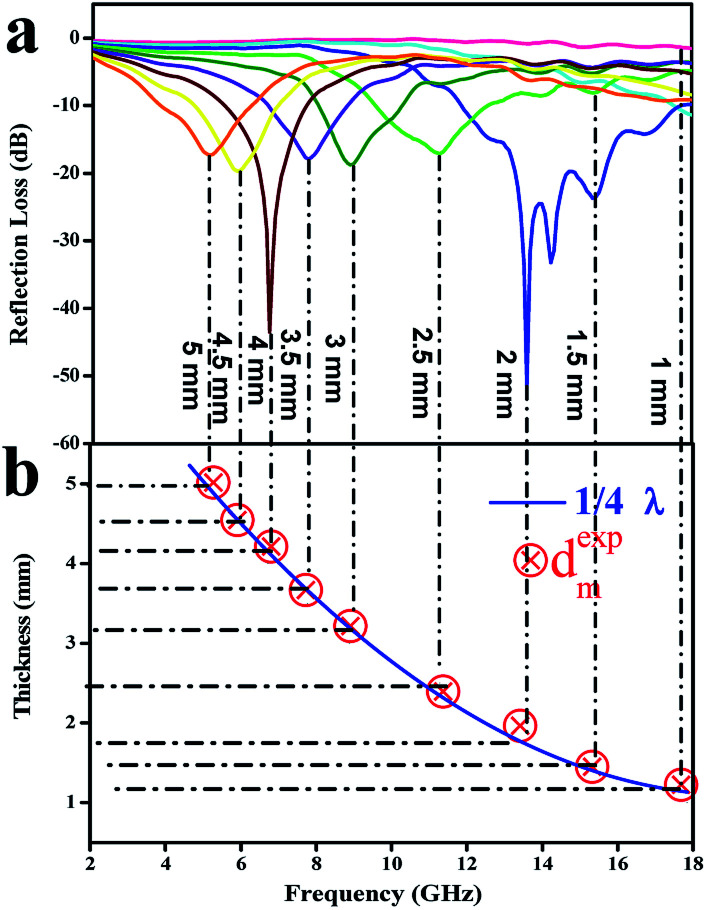
(a) Frequency dependence of RL curves for Fe_3_O_4_/WPC-600 composites with nine thicknesses; (b) plot of absorber thickness (*d*_m_) *versus* peak frequency (*f*_m_) for the Fe_3_O_4_/WPC-600 composites according to the *λ*/4 model.

As we know, the practical applications of microwave-absorbing materials are determined by the frequency of the absorption peak and the thickness of the microwave absorber. It is noteworthy that the frequency decreased with an increase in the absorber thickness (Fig. 11a), which can be explained by the quarter-wavelength matching theory (*λ*/4) according to eqn (11):^7,57^

11 *d*_m_ = *nλ*/4 = *nc*/4*f*_m_√*ε*_r_*μ*_r_ (*n* = 1, 3, 5,…)

where *d*_m_ is the absorber thickness, *f*_m_ is the frequency of the absorption peak, *c* is the velocity of light in a vacuum, and *λ* is the wavelength of the microwaves. According to the *λ*/4 model, if *f*_m_ and *d*_m_ satisfy the equation, the incident and reflected waves at the material interface are out of phase by 180°, with the result that the reflected waves at the air-absorber interface are cancelled totally.^58^ Obviously, the red circles are exactly situated around the *λ*/4 curve, which suggests that the relationship between the matching thickness and peak frequency for electromagnetic wave absorption obeys the *λ*/4 model (Fig. 11b). Furthermore, the quarter-wavelength matching theory is considered to significantly guide the choice of the thickness of an electromagnetic-wave absorber, once the required complex permeability and permittivity are attained.

**Scheme 2:**
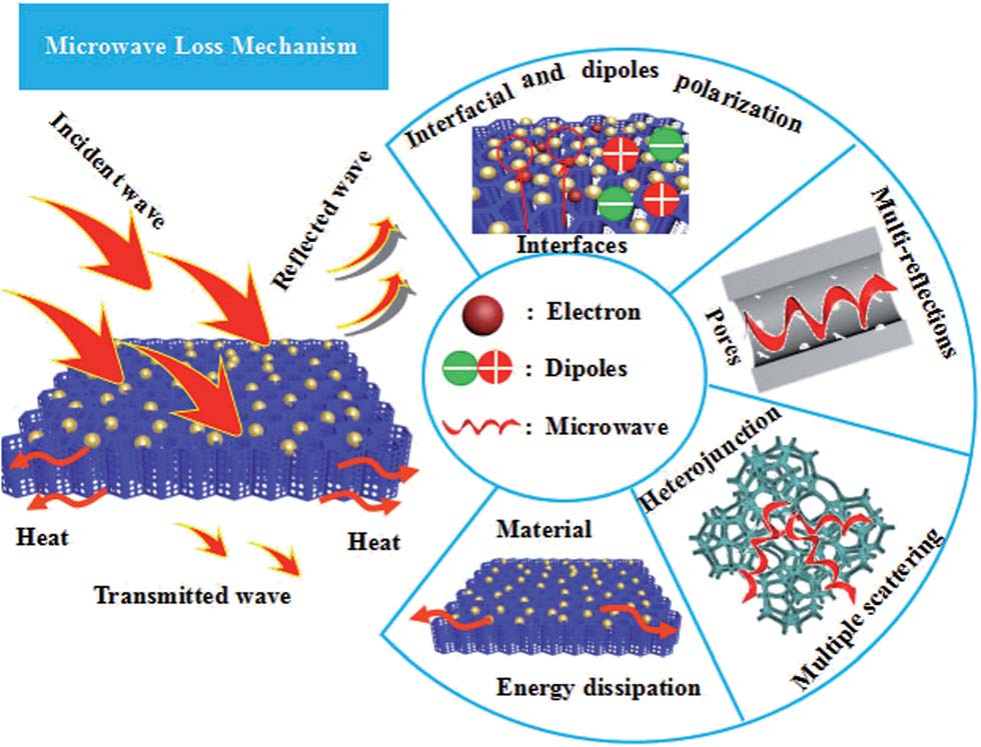
Plausible mechanism of microwave absorption by the fabricated Fe_3_O_4_/WPC-600 composites.

On the basis of the abovementioned analysis, the high electromagnetic-wave absorption by Fe_3_O_4_/WPC-600 could be understood *via* several proposed mechanisms, as shown in Scheme 2. When electromagnetic waves are incident on the surface of Fe_3_O_4_/WPC-600, some EM waves are immediately reflected owing to abundant free electrons on the surface.^59^

The remaining waves pass through the interface of the material because of perfect impedance matching. In this process, the EM waves are attenuated as a result of interfacial polarization as well as dipole polarization, which is due to the aggregation of bound charges at the interfaces and nanoparticles. Oxygen, in particular, as it is highly electronegative, can induce this kind of dipole polarization. When EM waves are transmitted into this material, the porous structure and defects will provide abundant active sites to induce multiple reflections and scattering of the electromagnetic waves and dissipate them as energy. After constant reflection and scattering, microwaves can be effectively absorbed by Fe_3_O_4_/WPC-600.

## Conclusion

In conclusion, a facile solvothermal route was developed for fabricating Fe_3_O_4_/WPC-600 composites as an improved microwave absorber. Fe_3_O_4_ nanoparticles were uniformly decorated on the surface of porous carbon derived from walnut shells without much aggregation or vacancies. In comparison with porous carbon and pure Fe_3_O_4_ nanoparticles, the Fe_3_O_4_/WPC-600 composites displayed improved microwave absorption performance owing to the synergistic effect between lightweight conductive porous carbon with dielectric loss and Fe_3_O_4_ nanoparticles with magnetic loss. The Fe_3_O_4_/WPC-600 composites exhibited excellent microwave absorption performance in that the maximum RL value reached −51.6 dB at a frequency of 13.6 GHz. Besides, the effective absorption (below −10 dB) bandwidth reached 5.8 GHz (from 11.9 to 17.7 GHz) at an absorber thickness of 2 mm. A study showed that crucial factors for efficient microwave absorbers are microwave attenuation and impedance matching. In addition, the relationship between the absorber thickness and peak frequency obeys the quarter-wavelength matching theory, which facilitates the choice of a matching thickness for microwave absorbers. Owing to their advantages of cheapness, light weight, broadband absorption, low matching thickness and strong absorption performance, the Fe_3_O_4_/WPC-600 composites are attractive candidates for the design and manufacture of new types of microwave-absorbing materials.

## Conflicts of interest

There are no conflicts to declare.

## Supplementary Material

RA-008-C8RA00913A-s001
