# Supplementary material for: Significant promotion of porous architecture and magnetic Fe3O4 NPs inside honeycomb-like carbonaceous composites for enhanced microwave absorption
Source: RSC Adv. 2018 May 23;8(34):19011–23. doi: 10.1039/c8ra00913a (PMC9080601; doi:10.1039/c8ra00913a)
Supplement: RA-008-C8RA00913A-s001 [file RA-008-C8RA00913A-s001.pdf]

Electronic Supplementary Material (ESI) for RSC Advances.
This journal is © The Royal Society of Chemistry 2018

Supporting Information

Significant promotion of porous architecture and magnetic Fe_3O_4 NPs inside honeycomb-like carbonaceous composites for enhanced microwave absorption

Shengshuai Gao [a], Qingda An [a,*], Zuoyi Xiao [a], Shangru Zhai [a,*], Zhan Shi [b]

[a] *Faculty of Light Industry and Chemical Engineering, Dalian Polytechnic University, Dalian 116034, China*

[b] *State Key Laboratory of Inorganic Synthesis and Preparative Chemistry, College of Chemistry, Jilin University, Changchun 130012, China*

*Corresponding authors.

E-mail: anqingdachem@163.com (Q.-D. An); zhaisrchem@163.com (S.-R. Zhai)

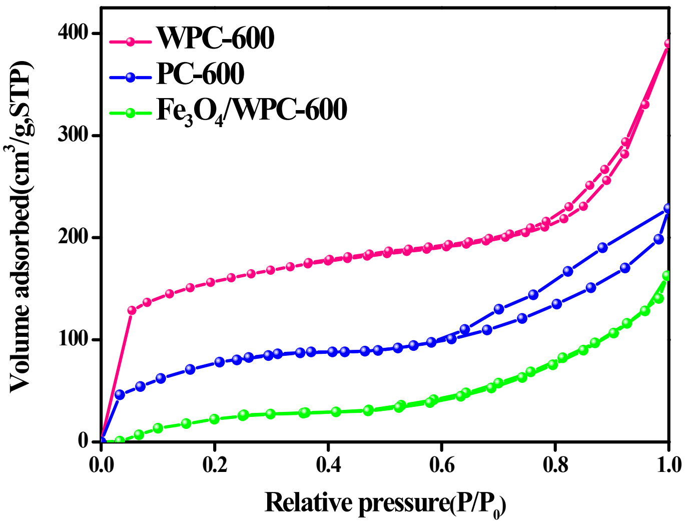

Fig. S1. Nitrogen adsorption - desorption isotherms of as-prepared samples under different conditions.

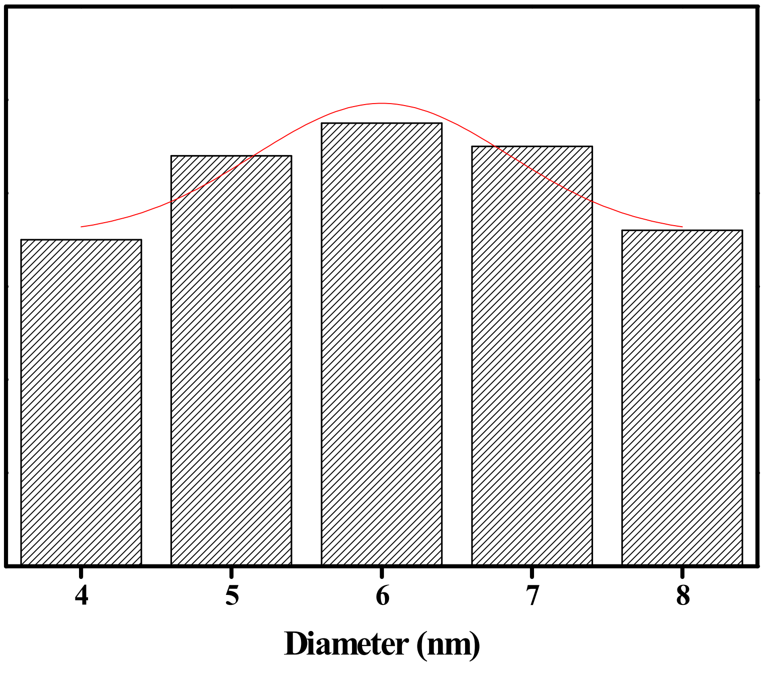

Fig. S2. Size distributions of Fe_3O_4 nano-particles for Fe_3O_4/WPC-600 composites.

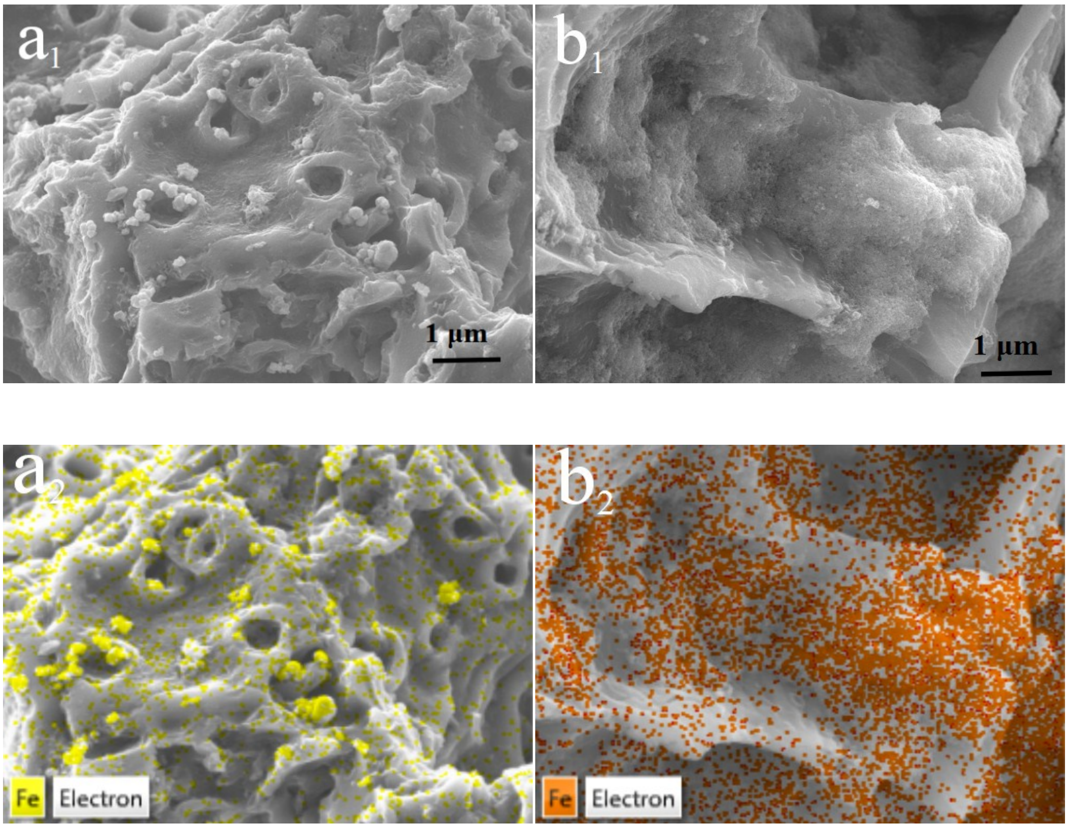

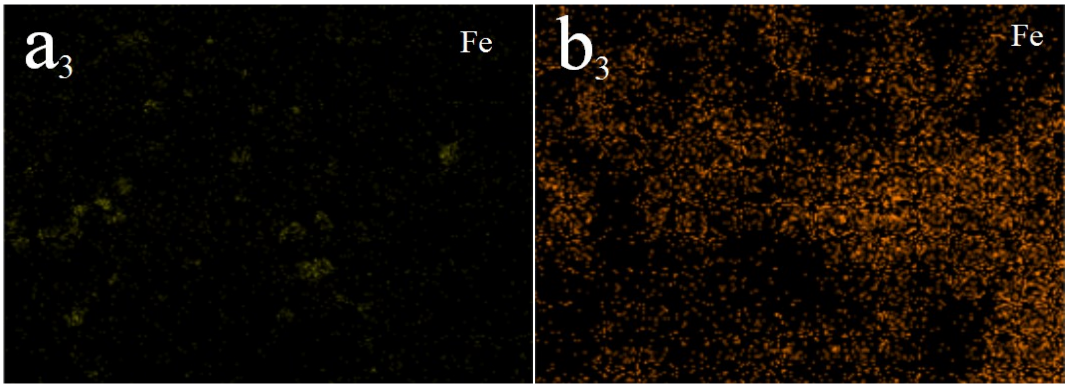

Fig. S3 SEM images of 0.5-Fe_3O_4/WPC-600 (a_1), 2-Fe_3O_4/WPC-600 (b_1), EDS elemental mapping images of 0.5-Fe_3O_4/WPC-600 (a_2 and a_3) and 2-Fe_3O_4/WPC-600 (b_2 and b_3)

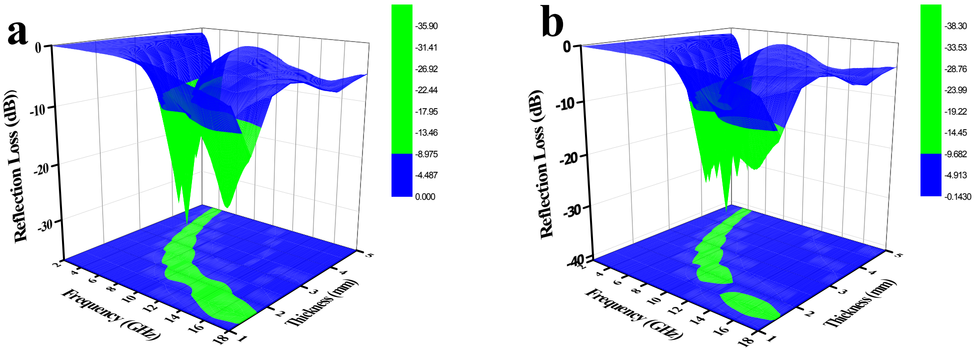

Fig. S4 Three-dimension images of calculated RL values of (a) 0.5-Fe_3O_4/WPC-600 and (b) 2-Fe_3O_4/WPC-600.

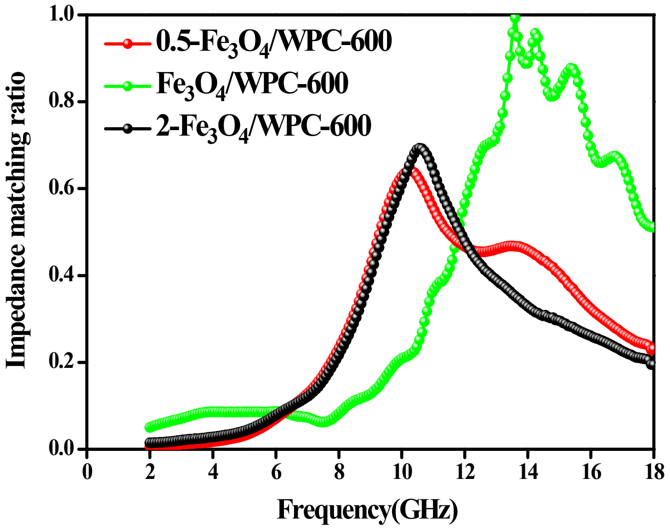

Fig. S5 The modulus of normalized input impedance of the products.

Table S1 The conductivity σ of samples.

Sample	PC-600	WPC-600	0.5-Fe_3O_4/WPC-600	Fe_3O_4/WPC-600	2-Fe_3O_4/WPC-600
σ(S/m)	1.27×10^{-7}	6.23×10^{-5}	7.45×10^{-5}	1.16×10^{-4}	4.06×10^{-4}
